# Skeletal Muscle: A Significant Novel Neurohypophyseal Hormone-Secreting Organ

**DOI:** 10.3389/fphys.2018.01885

**Published:** 2019-01-08

**Authors:** Sergio Adamo, Eva Pigna, Rosamaria Lugarà, Viviana Moresi, Dario Coletti, Marina Bouché

**Affiliations:** ^1^Section of Histology & Medical Embryology, Department of Anatomical, Histological, Forensic and Orthopedic Sciences, Interuniversity Institute of Myology, Sapienza University of Rome, Rome, Italy; ^2^Sorbonne Université, CNRS UMR 8256–INSERM ERL U1164, Biological Adaptation and Aging B2A, Paris, France

**Keywords:** vasopressin, oxytocin, myogenesis, muscle regeneration, satellite cell, muscle atrophy and hypertrophy, anabolizing agents

Vasopressin (arg^8^-vasopressin) and oxytocin are closely related nonapeptide hormones, synthesized as pre-hormones in the magnocellular neurons of the paraventricular and supraoptic nuclei of the hypothalamus. Vasopressin and oxytocin are secreted in response to a variety of physiological stimuli, serving such different functions as controlling water balance, milk ejection, uterine contraction, mood, and parental behavior (Lechan and Toni, 2000; Costa et al., 2014a).

## Muscle as a Target of Neurohypophyseal Hormones

Skeletal muscle or myogenic precursors have never been considered, until recently, as targets of neurohypophyseal hormones. However, very early evidence was provided by Wakelam and collaborators, who showed some mild effects of vasopressin on carbohydrate metabolism in myoblasts (Wakelam and Pette, 1982; Wakelam et al., 1987). These initial observations were corroborated by later studies showing that stimulation of primary chick embryo myoblasts or murine L6 and L5 myogenic cell lines with vasopressin, oxytocin, their analogs and antagonists resulted in the structure- and concentration-dependent activation of phospholipase C (PLC) signaling, the stimulation of myogenic differentiation, and the hypertrophy of newly formed muscle fibers (Teti et al., 1993; Nervi et al., 1995). Worth noting, PLC activates Protein Kinase C family members, among which the theta isoform plays an important role in both muscle differentiation and disease (Marrocco et al., 2014, 2017; Lozanoska-Ochser et al., 2018). The potent pro-myogenic effect of vasopressin was further characterized in myogenic cells cultured in a serum-free medium, a “clean” experimental model that allowed us to establish that L6 myogenic cells express the vasopressin V1a receptor (V1a-R) and that vasopressin elicits a complex signal transduction response in these cells (Minotti et al., 1998; Scicchitano, 2002, 2005; Naro et al., 2003; Toschi et al., 2011; Costa et al., 2014b). Moreover, it was found that V1a-R expression is modulated during the differentiation of L6 cells, probably in a post-translational manner (Alvisi et al., 2008).

Studies from other laboratories highlighted the presence of functional oxytocin receptors (OT-R) in human myoblasts derived from postnatal satellite cells (Breton et al., 2002), and in C2C12 myogenic cells which respond to oxytocin by activating the calcium–CaMKK–AMPK pathway (Lee et al., 2008). A recent *in vitro* study showed that C2C12 myoblasts express not only OT-R but also oxytocin and that the expression of both products increases upon myogenic differentiation of the cells (Berio et al., 2017). Furthermore, myotubes treated with 17β-estradiol overexpress oxytocin and OT-R genes by approximately 3- and 29-fold, respectively (Berio et al., 2017).

While cell cultures provide useful models to define, under controlled conditions, the effects of vasopressin or oxytocin on myogenic differentiation at the molecular level, *in vivo* data support the idea that both neurohypophyseal hormones play physiological roles in skeletal muscle. Indeed, a role for neurohypophyseal hormones in prenatal muscle development was first suggested by the presence of immunoreactive vasopressin in human fetal and neonatal skeletal muscle (Smith et al., 1992). Moreover, a >120-fold increase in oxytocin expression was observed in bovine muscle during early to mid-fetal calf development, coincident with active myofiber formation (De Jager et al., 2011), findings in keeping with the above reported *in vitro* data.

In an *in vivo* murine experimental model, we reported that V1a-R expression, measurable under basal conditions in muscle, was strongly up-regulated in the early phase of regeneration (9 h after injury) and gradually decreased in the following days, along with the regeneration process. In this model, vasopressin administration promoted muscle regeneration. Furthermore, overexpression of V1a-R in muscle sufficed to dramatically enhance post-injury muscle regeneration, without administering exogenous vasopressin (Toschi et al., 2011). In an *in vivo* mouse model of tumor necrosis factor (TNF)-inhibited muscle regeneration, the administration of vasopressin rescued the inhibitory effect of TNF, likely through a mechanism involving the modulation of HSP70 levels (Moresi et al., 2009). Again, TNF-mediated muscle atrophy was rescued by the overexpression of the V1a-R *in vivo* (Costa et al., 2014b).

Altogether, the *in vivo* evidence supports the notion that both vasopressin and oxytocin have potent effects, in the development, regeneration and homeostasis of skeletal muscle.

Further insights came from studies conducted in aged mice (Elabd et al., 2014). The authors focused on the reduced muscle regeneration and muscle atrophy (sarcopenia) occurring in aging. While it is known that aging is accompanied by reduced physiological levels of sex steroids, the authors found that circulating oxytocin level is also reduced. Interestingly, satellite cells from aged animals exhibited a significantly lower OT-R expression than those from young animals. Moreover, comparing young and aged mice treated with an oxytocin selective antagonist or with exogenous oxytocin, respectively, the authors demonstrated that oxytocin is required for efficient muscle regeneration (Elabd et al., 2014). The impaired muscle regeneration of aged mice was shown to depend primarily upon reduced proliferation of satellite cells, a phenomenon rescued by exogenous oxytocin administration. The oxytocin effect on satellite cell proliferation was reported to be mediated by the MAPK/ERK pathway (Elabd et al., 2014). In line with the above findings, muscle regeneration in oxytocin KO mice was severely compromised. Young oxytocin KO mice displayed a premature decline in muscle regeneration, as well as muscle fibrosis and fat infiltration, showing a muscle phenotype characteristic of sarcopenia (Elabd et al., 2014).

Therefore, both neurohypophyseal hormones appear to regulate positively muscle homeostasis in different models: oxytocin, in the aging and KO mouse models (Elabd et al., 2014); and vasopressin, in injured muscle or TNF-induced muscle wasting (Moresi et al., 2009; Toschi et al., 2011; Costa et al., 2014b).

The apparent overlapping between the observed effects of the two neurohypophyseal hormones in the regulation of muscle differentiation and trophism may depend on the fact that both V1a-R and OT-R cross-bind their ligands, albeit with different affinities (Barberis et al., 1998; Gupta et al., 2008). Furthermore, the results obtained in the myogenic L6 cell line used as an experimental tool to show the effect of neurohypophyseal hormones probably depend on a peculiar expression of the receptors for these hormones, in comparison to other myogenic cell types.

## Muscle Tissue as a Source of Vasopressin and Oxytocin

Interestingly, the possibility that myogenic cells express one of the neurohypophyseal hormones, as suggested by the pioneering results of Smith in prenatal human muscle for vasopressin (Smith et al., 1992), was recently proposed again (Berio et al., 2017), with regard to oxytocin and OT-R. Based on these observations, muscle can thus be added to the list of previously unrecognized sites of oxytocin expression, such as testes, ovaries, heart and lungs (Assinder et al., 2000; Jankowski et al., 2004; Kiss and Mikkelsen, 2005; Gutkowska and Jankowski, 2012). Further results supporting this hypothesis, were obtained in studies aimed to investigate the mechanisms triggered by the administration of steroids used to increase muscle mass in livestock farming. Cattle regularly treated with anabolizing agents displayed dramatically enhanced oxytocin mRNA expression in skeletal muscle, accompanied by a ~50-fold higher level of circulating oxytocin (De Jager et al., 2011). Intriguingly, the authors provided evidence that the hypertrophying effect of anabolic steroids is prevalently mediated by OT-R signaling. Furthermore, in a more recent study, Divari reported that serum levels of oxytocin increased dramatically in cattle regularly treated with 17β-estradiol, but not with either dexamethasone or placebo. This administration of 17β-estradiol also resulted in increased (33-fold) skeletal muscle expression of the oxytocin-precursor mRNA (Divari et al., 2013). Increased expression of the oxytocin-precursor mRNA was also found in the muscle of sheep subjected to chronical treatment with a combination of 17β-estradiol and the synthetic androgen trenbolone acetate (TBA). Also the circulating oxytocin level increased in steroid-treated sheep compared to placebo-treated controls (Kongsuwan et al., 2012). Together, the above studies indicate a correlation between the steroid-induced muscle hypertrophy and the increased expression of both oxytocin and OT-R in skeletal muscle.

The mechanisms underlying regulation by steroids of oxytocin expression remain to be fully elucidated. The oxytocin promoter does not possess a classical Estrogen Response Element, whereas it has a high affinity binding-site for nuclear orphan receptors/estrogen related receptor alpha (ERRα). Koohi reported that the estrogen dependent control of the oxytocin promoter is independent of classical Estrogen Receptor (ER) binding, but requires a functional ERRα (Koohi et al., 2005). These authors further demonstrated that the estrogenic stimulation of the OT-R occurs through the ERK/MAPK-mediated stimulation of the transcriptional activity of ERRα. The up-regulation of oxytocin expression by this non-classical mechanism may, in turn, sustain an autocrine feed-forward oxytocin/OT-R loop which amplifies the response to oxytocin, as shown in bone (Colaianni et al., 2012; Berio et al., 2017).

Worth noting, exercise (i.e., muscle contraction) represents a physiological stimulus increasing the levels of circulating neurohypophyseal hormones, as well as their expression (or the expression of their receptors) in several tissues beside skeletal muscle (Martins et al., 2005). Based on several studies in man and other mammals, it is clear that exercise induces a five- fold increase in the circulating levels of vasopressin (Melin et al., 1980; Convertino et al., 1981; Alexander et al., 1991). This increase, associated to the beneficial effects of exercise on muscle homeostasis, suggests a model whereby physical activity stimulates muscle secretion of the neurohypophyseal hormones and induces a generalized sensitization to these factors through the up-regulation of their receptors in various districts. An exercise-mediated increase in vasopressin and/or oxytocin could ultimately contribute to maintaining muscle homeostasis and add to the additional benefits of exercise, including an increased life span and general well-being.

## Final Remarks

The *in vitro* and *in vivo* studies discussed above suggest that skeletal muscle is a target of neurohypophyseal hormones, which regulate muscle homeostasis and function in both physiological and pathological conditions. On the other hand, skeletal muscle has been recognized as the source of a wide range of circulating factors, namely myokines, which regulate a number of different functions with paracrine or endocrine mechanisms (Pedersen and Febbraio, 2008; Hoffmann and Weigert, 2017). In the light of its abundance in the organism, skeletal muscle may thus be regarded as the largest endocrine gland in the body. In this article, we discuss *in vitro* and *in vivo* studies showing that oxytocin is synthesized by muscle and that its secretion significantly contributes to the level of circulating hormones. This activity appears to be central to the mechanisms which regulate muscle homeostasis, to contribute to muscle hypertrophic responses and to be altered in atrophic conditions. As a whole, this evidence suggests that OT and AVP be considered as potential myokines. In addition, these considerations suggest a potential therapeutic use of these molecules, along with more selective and potent analogs, in atrophic and muscle wasting conditions, such as sarcopenia and cachexia, and as a tool in adjuvant therapies against muscular dystrophies and neuromuscular diseases.

## Author Contributions

SA, MB drafted the manuscript and approved the final version. EP, RL contributed to manuscript writing. DC, VM provided important interpretations, critically revised the manuscript. All authors provided final approval of the opinion content.

### Conflict of Interest Statement

The authors declare that the research was conducted in the absence of any commercial or financial relationships that could be construed as a potential conflict of interest.
